# Predictive Value of C-Reactive Protein/Albumin Ratio (CAR) for Malnutrition and Sarcopenia in Acute Ischemic Stroke Patients

**DOI:** 10.3390/jcm14196804

**Published:** 2025-09-26

**Authors:** Hasan Dogan, Sugra Simsek, Ahmet Hakan Bayram, Aydan Topal, Mehlika Berra Pamuk, Ozkan Ozmuk, Nedim Ongun, Cetin Kursad Akpinar

**Affiliations:** 1 Department of Neurology, Samsun University Faculty of Medicine, Samsun 55020, Turkey; 2 Neurology Clinic, Samsun Training And Research Hospital, Samsun 55020, Turkey; 3 Neurology Clinic, Antalya City Hospital, Antalya 07000, Turkey

**Keywords:** C-reactive protein/albumin ratio (CAR), Malnutrition, sarcopenia, acute ischemic stroke, biomarkers, nutritional risk

## Abstract

**Background/Objective:** Malnutrition and sarcopenia are common complications after ischemic stroke and have a negative impact on prognosis. The C-reactive protein/albumin ratio (CAR) reflects both inflammation and nutritional status, but its predictive role in this setting has not been widely studied. This study aimed to investigate the predictive value of CAR (C-reactive protein/albumin ratio) for malnutrition risk and probable sarcopenia in patients with ischemic stroke. **Methods:** In this prospective observational study, 197 patients with acute ischemic stroke were evaluated. Patients with chronic renal or hepatic failure, malignancy, active infection, and hand disability preventing grip strength measurement were excluded. Demographic data (age, sex), vascular risk factors, the NIHSS score, and laboratory parameters were recorded. The nutritional status of patients was assessed using the Nutritional Risk Screening-2002 (NRS-2002), and sarcopenia risk was evaluated with the SARC-F questionnaire. Handgrip strength was measured in patients with high SARC-F scores to define probable sarcopenia. CAR was calculated from serum CRP and albumin levels. Logistic regression was applied to identify independent predictors, and receiver operating characteristic (ROC) analyses were performed to determine the discriminatory ability and cut-off values of CAR. The nutritional status of patients admitted to the neurology clinic with acute ischemic stroke was assessed using the Nutritional Risk Screening-2002 (NRS-2002), and sarcopenia risk was evaluated with the SARC-F questionnaire. Handgrip strength was measured in patients with high SARC-F scores to define probable sarcopenia. CAR was calculated from serum CRP and albumin levels. Logistic regression and receiver operating characteristic (ROC) analyses were performed. **Results:** Malnutrition risk was identified in 32.5% of patients, and probable sarcopenia was identified in 19.3% of patients. ROC analysis showed that CAR had acceptable discriminatory power for both conditions. In multivariate analysis, CAR was consistently identified as an independent predictor of malnutrition risk and possible sarcopenia. ROC analysis for malnutrition risk showed an AUC of 0.750 (cut-off: 0.306; sensitivity 68.8%; specificity 75.2%). In regression analysis, CAR (OR = 2.13; 95% CI: 1.39–3.26; *p* < 0.001), age (OR = 1.05; 95% CI: 1.02–1.09; *p* = 0.003), and NIHSS (OR = 1.11; 95% CI: 1.01–1.23; *p* = 0.026) were independent predictors. For probable sarcopenia, ROC analysis revealed an AUC of 0.814 (cut-off: 0.320; sensitivity 81.6%; specificity 71.7%). Multivariate analysis identified CAR (OR = 1.73; 95% CI: 1.19–2.52; *p* = 0.004), age (OR = 1.11; 95% CI: 1.05–1.18; *p* < 0.001), and NIHSS (OR = 1.19; 95% CI: 1.05–1.35; *p* = 0.007) as independent predictors. **Conclusions:** CAR was identified as an independent predictor of both malnutrition risk and probable sarcopenia in ischemic stroke patients. CAR may serve as a reliable biomarker for early nutritional and functional risk stratification in clinical practice.

## 1. Introduction

Despite recent advances in stroke treatment, it remains one of the leading causes of mortality and disability worldwide [1]. According to data from the World Health Organization, one in four adults will experience a stroke at least once in their lifetime [2]. Consequently, stroke continues to represent a major global public health challenge.

One of the most common complications in the post-stroke period is malnutrition. Up to 60% of patients with ischemic stroke may be malnourished or at risk of malnutrition [3]. Furthermore, 16–32% of patients have been reported to present with poor nutritional status at the time of hospital admission for stroke [4,5,6]. Sarcopenia, characterized by progressive loss of muscle mass and strength and resulting impairment in muscle function, represents an important problem in stroke patients. The prevalence of post-stroke sarcopenia has been reported to reach up to 50%. Additionally, post-stroke immobilization, dysphagia, and inflammatory processes further contribute to the development of sarcopenia [7].

Malnutrition has a significant impact on stroke prognosis and is associated with prolonged hospital stays, increased mortality, higher complication rates, and poorer functional outcomes [8]. Similarly, patients with sarcopenia have longer hospital stays, poorer functional outcomes, and more limited recovery after rehabilitation [7,9]. Therefore, the early identification and prevention of both malnutrition and sarcopenia are critical components of comprehensive stroke management.

A systemic inflammatory response occurs in the acute phase of stroke [10]. This inflammation triggers mechanisms known to reduce albumin levels, including decreased hepatic synthesis, increased vascular permeability, and enhanced protein catabolism [11]. Albumin, a negative acute-phase reactant, is also commonly used as a marker of nutritional status [6]. In stroke patients, low serum albumin levels have been associated with poor functional outcomes and increased mortality [12]. In contrast, C-reactive protein (CRP), a positive acute-phase reactant, is rapidly upregulated by pro-inflammatory cytokines such as IL-6 and serves as a strong indicator of systemic inflammation. Elevated CRP levels have been consistently associated with greater stroke severity, poor functional outcomes, and increased mortality in stroke patients [13,14]. C-reactive protein (CRP), a positive acute-phase reactant, is a strong indicator of inflammation, and its elevation has been associated with poor functional outcomes and mortality in stroke patients [12].

The CRP-to-albumin ratio (CAR), derived from the combined evaluation of CRP and albumin levels, may serve as a biomarker reflecting both systemic inflammation and nutritional status. Elevated CAR levels have been associated with increased mortality and poor prognosis in cardiovascular diseases, critically ill patients, and oncological conditions [15,16,17,18]. These findings suggest that CAR may represent a powerful, practical, and easily applicable parameter across various clinical conditions.

The aim of this study is to investigate whether the CRP-to-albumin ratio (CAR) can predict nutritional status and the risk of sarcopenia in patients with ischemic stroke, thereby highlighting its potential as a simple, cost-effective, and reliable biomarker for use in clinical practice.

## 2. Material and Methods

### 2.1. Study Design and Population

This prospective observational study enrolled all consecutive patients with acute ischemic stroke who were admitted to two tertiary neurology clinics between March and June 2025. This study was conducted on patients followed with a diagnosis of acute ischemic stroke in two tertiary neurology clinics. Inclusion criteria were age ≥ 18 years, confirmed diagnosis of acute ischemic stroke, and ability to provide informed consent (by patient or legal representative). Exclusion criteria were as follows: chronic renal and/or hepatic failure, history of malignancy, active infection, and any hand disability that would prevent adaptation to handheld dynamometry. Patients with missing data or lost to follow-up were also excluded. The study was approved by the Samsun University Medical School Non-Interventional Clinical Trials Ethics Committee (GOKAEK 2025/5/20). All participants provided written informed consent prior to participation. All assessments were performed by a trained neurologist following a standardized protocol. Since each patient was evaluated by a single assessor, no inter-rater reliability analysis was required. Demographic and clinical data were recorded at admission, including age, sex, vascular risk factors (hypertension, diabetes mellitus, hyperlipidemia, atrial fibrillation, and coronary artery disease), body mass index (BMI) and smoking status. These comorbidities were systematically documented because previous studies have shown their association with impaired nutritional status and sarcopenia [19,20]. Stroke severity was assessed using the NIHSS score at admission. Body weight and height measurements were taken within the first 24 h after admission. Nutritional status was assessed at admission using Nutritional Risk Screening 2002 (NRS-2002), a validated tool recommended by the European Society for Clinical Nutrition and Metabolism (ESPEN). This screening tool evaluates disease severity, age, recent weight loss, BMI, and oral intake; a score of ≥3 indicates risk of malnutrition [21]. The NRS-2002 test, which was performed upon admission to determine the risk of malnutrition, was recorded in all patients. The NRS-2002 test was recorded for all patients at the time of admission to assess malnutrition risk. NRS-2002 is a test that questions the severity of the disease as well as age, weight loss, body mass index and oral intake. Patients with a score of 3 or higher are considered at risk of malnutrition. The European Working Group on Sarcopenia in Older People 2 (EWGSOP2) recommends the use of the SARC-F questionnaire as a screening tool for sarcopenia risk, which obtains personal reports on symptoms of sarcopenia [22]. SARC-F is a 5-item questionnaire easily used in clinical settings, with a score of 4 or higher indicating sarcopenia risk [23]. Muscle strength was assessed using a calibrated handheld dynamometer. Two trials were performed for each hand, and the best result from the strongest hand was used. Patients with reduced handgrip strength, based on EWGSOP2 sex-specific cut-off values, were classified as having probable sarcopenia. Patients with SARC-F < 4 or normal grip strength were classified as non-sarcopenic.

Venous blood samples were collected from all patients within the first 24 h following hospital admission. Serum albumin (g/L) and C-reactive protein (CRP, mg/L) levels were analyzed. The CRP-to-albumin ratio (CAR) was calculated by dividing the serum CRP

### 2.2. Statistical Analysis

Data were analyzed using IBM SPSS Statistics Version 22.0 (SPSS Inc., Chicago, IL, USA). The distribution of continuous variables was assessed using the Kolmogorov–Smirnov test. Variables with normal distribution were presented as mean ± standard deviation, while non-normally distributed variables were expressed as median (minimum–maximum). Categorical variables were presented as frequencies and percentages (%). For comparisons between groups, the chi-square test or Fisher’s exact test (when appropriate) was used for categorical variables. For continuous variables, the independent samples *t*-test or Mann–Whitney U test was applied depending on the distribution. To identify factors predicting the risk of malnutrition and sarcopenia, univariate logistic regression analysis was first performed, followed by multivariate logistic regression analysis for variables found to be significant. Variables that were significant in univariate analysis, as well as clinically relevant covariates (age, sex, BMI, and NIHSS), were included in the multivariate models to adjust for potential confounding effects. Multicollinearity among the independent variables was assessed using the variance inflation factor (VIF) and tolerance values. All variables had VIF < 2 and tolerance > 0.1, indicating no collinearity (Supplementary Materials). The results were reported as odds ratios (OR) with 95% confidence intervals (CI). Additionally, the optimal cutoff value for CAR was determined using the receiver operating characteristic (ROC) curve analysis. The area under the curve (AUC) was calculated, and sensitivity and specificity values were reported. To explore the overlap between malnutrition and probable sarcopenia, patients were cross-classified into four groups (none, malnutrition only, probable sarcopenia only, both). CAR distributions across these groups were compared using the Kruskal–Wallis test. In addition, correlations between CAR and NRS-2002 scores as well as handgrip strength were analyzed using Spearman’s correlation coefficient. A *p*-value < 0.05 was considered statistically significant.

## 3. Results

A total of 197 patients were included in the study, with a mean age of 69.1 ± 12.4 years; 111 of them (56.3%) were male. Based on the NRS-2002 assessment, 64 patients (32.5%) were identified as being at risk of malnutrition, while probable sarcopenia (SARC-F ≥ 4 plus sex-specific low handgrip strength) was detected in 38 patients (19.3%). Notably, 36 of the 38 patients with probable sarcopenia also belonged to the malnutrition-risk group. CAR levels showed a graded increase across the four strata (no risk, malnutrition risk only, probable sarcopenia only, both; Kruskal–Wallis χ^2^ = 42.25, *p* < 0.001), with the highest levels observed in patients with both malnutrition risk and probable sarcopenia. In addition, CAR correlated positively with NRS-2002 scores (ρ = 0.426, *p* < 0.001) and inversely with handgrip strength (ρ = −0.392, *p* < 0.001), supporting its central role across both outcomes.

**CAR and Malnutrition Risk**

The demographic and clinical characteristics of patients with and without malnutrition risk are summarized in Table 1. Patients in the malnutrition-risk group were significantly older (*p* < 0.001), had higher NIHSS scores (*p* = 0.001), and had a higher prevalence of atrial fibrillation (AF) (*p* = 0.002). Additionally, they had significantly lower BMI values (*p* < 0.001) and higher CAR levels (*p* < 0.001). No significant differences were observed between the groups in terms of sex, diabetes mellitus (DM), hypertension (HT), coronary artery disease (CAD), hyperlipidemia (HL), or smoking status.

**Figure 1:**
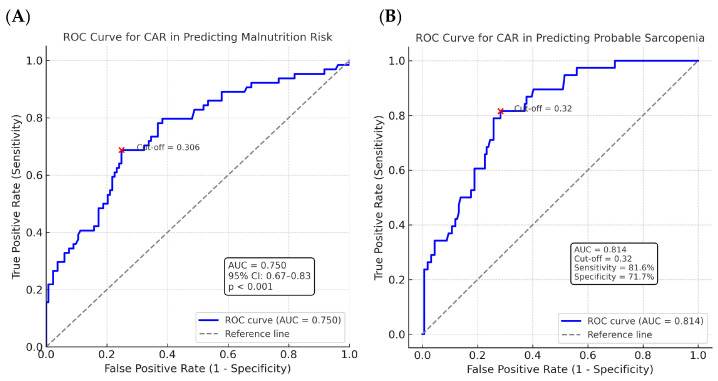
Receiver operating characteristic (ROC) curves of the C-reactive protein/albumin ratio (CAR) for predicting (**A**) malnutrition risk and (**B**) probable sarcopenia in patients with ischemic stroke. The optimal cut-off values are indicated on each curve.

The performance of CAR in predicting the risk of malnutrition was evaluated using a receiver operating characteristic (ROC) curve analysis. The area under the curve (AUC) was found to be 0.750 (95% CI: 0.67–0.83; *p* < 0.001). The optimal cutoff value for CAR was determined to be 0.306, with a sensitivity of 68.8% and a specificity of 75.2% at this threshold (Figure 1).

Univariate and multivariate logistic regression analyses were conducted to identify factors associated with the risk of malnutrition. In the univariate analysis, CAR (OR = 2.47, 95% CI: 1.65–3.71, *p* < 0.001), age (OR = 1.06, 95% CI: 1.03–1.10, *p* < 0.001), NIHSS score (OR = 1.15, 95% CI: 1.06–1.25, *p* < 0.001), AF (OR = 2.78, 95% CI: 1.48–5.24, *p* = 0.001), and BMI (OR = 0.86, 95% CI: 0.80–0.94, *p* < 0.001) were all significantly associated with malnutrition risk. In the multivariate analysis, CAR (OR = 2.13, 95% CI: 1.39–3.26, *p* < 0.001), age (OR = 1.05, 95% CI: 1.02–1.09, *p* = 0.003), NIHSS score (OR = 1.11, 95% CI: 1.01–1.23, *p* = 0.026), and BMI (OR = 0.89, 95% CI: 0.82–0.97, *p* = 0.001) remained independent predictors (Table 2).

**CAR and Probable Sarcopenia**

Forty-seven patients (23.9%) had a SARC-F score ≥ 4, and among them, 38 (19.3% of the total cohort) were classified as probable sarcopenia based on low handgrip strength. Compared with those without probable sarcopenia, affected patients were significantly older (*p* < 0.001), had higher NIHSS scores (*p* = 0.007), higher CAR levels (*p* < 0.001), and lower BMI (*p* = 0.004) and smoking prevalence (*p* = 0.027) (Table 3) Probable sarcopenia, as determined by the handgrip strength test, was identified in 38 patients (19.3%). In the probable sarcopenia group, age (78.47 ± 9.33 vs. 66.90 ± 12.35 years, *p* < 0.001), NIHSS score (6.08 ± 4.10 vs. 4.28 ± 3.63, *p* = 0.007), and CAR levels (1.81 ± 2.01 vs. 0.47 ± 0.89, *p* < 0.001) were significantly higher, while smoking prevalence (7.9% vs. 20.1%, *p* = 0.027) and BMI (26.72 ± 4.75 vs. 28.64 ± 4.93 kg/m^2^, *p* = 0.004) were significantly lower compared to patients without probable sarcopenia (Table 3).

The performance of CAR in predicting probable sarcopenia was assessed using ROC curve analysis. The AUC was found to be 0.814. The optimal cutoff value was determined to be 0.32, which yielded a sensitivity of 81.6% and a specificity of 71.7% (Figure 1).

In univariate logistic regression analyses, age (OR = 1.13, 95% CI: 1.07–1.18, *p* < 0.001), NIHSS (OR = 1.12, 95% CI: 1.03–1.23, *p* = 0.010), BMI (OR = 0.92, 95% CI: 0.85–0.99, *p* = 0.035), smoking (OR = 0.25, 95% CI: 0.07–0.87, *p* = 0.030) and CAR (OR = 1.96, 95% CI: 1.45–2.64, *p* < 0.001) were found to be significantly associated with probable sarcopenia. In multivariate analysis, age (OR = 1.12, 95% CI: 1.05–1.18, *p* < 0.001), NIHSS (OR = 1.16, 95% CI: 1.04–1.31, *p* = 0.011), and CAR (OR = 1.76, 95% CI: 1.22–2.53, *p* = 0.003) were identified as independent predictors (Table 4). Accordingly, each one-year increase in age was associated with a 12% increase in the risk of probable sarcopenia; each one-point increase in NIHSS score increased the risk by 16%; and higher CAR levels were associated with an approximately 1.7-fold increased risk.

Overall, these findings demonstrate that CAR consistently serves as a strong independent predictor of both malnutrition risk and probable sarcopenia, acting as a shared biomarker that reflects post-stroke nutritional status and decline in muscle strength.

## 4. Discussion

In this study, the relationship between the CAR and the risk of malnutrition and sarcopenia in patients with ischemic stroke was investigated. Our findings demonstrated that CAR is an independent predictor of both conditions. Notably, ROC analyses revealed that CAR had strong discriminatory power in predicting both nutritional risk and probable sarcopenia. In multivariate analyses, elevated CAR was associated with an approximately twofold increased risk of malnutrition and a 1.7-fold increased risk of probable sarcopenia. These results suggest that CAR may serve not only as a prognostic marker but also as a reliable biomarker for assessing nutritional status and muscle health. While previous studies in the literature have primarily focused on the association between CAR and mortality or functional outcomes, the current study’s focus on malnutrition and sarcopenia provides a novel and important perspective.

The risk of malnutrition increases after stroke due to factors such as dysphagia, impaired consciousness, and lack of cooperation [24]. Malnutrition is known to have a negative effect on mortality, length of hospital stays, and functional recovery [24,25]. Therefore, the European Society for Clinical Nutrition and Metabolism ESPEN recommends that all stroke patients undergo a nutritional assessment within the first 48 h of hospital admission [24].

Reported prevalence rates of post-stroke malnutrition vary widely in the literature. In a review conducted by Foley et al., these rates were found to range from 6.1% to 62% [26]. This wide range is thought to be from differences in assessment methods and the timing of measurements. Crary et al. reported that 26.3% of 76 stroke patients presented with poor nutritional status at admission [4]. Similarly, another study conducted in a neurology department reported a 30% risk of malnutrition upon admission [27]. In our study, the risk of malnutrition was identified in 32.5% of patients, a result consistent with previous reports.

Cerebral arterial occlusion leads to hypoxia, resulting in the accumulation of reactive oxygen species and subsequent tissue necrosis. Necrotic tissue triggers an inflammatory response that initially begins locally; however, with the release of proinflammatory cytokines such as IL-1 and IL-6, this response becomes systemic and is accompanied by disruption of the blood–brain barrier [28]. Through the action of IL-6, CRP synthesis from hepatocytes is stimulated, and elevated CRP levels are frequently observed after stroke [29]. High CRP levels have been associated with poor functional outcomes, increased mortality, and stroke recurrence [30]. Conversely, inflammation leads to reduced albumin levels, which are related to both decreased hepatic synthesis and increased catabolism [31]. Hypoalbuminemia has also been linked to adverse functional outcomes and mortality after stroke [12].

This biological process also provides a favorable environment for malnutrition and muscle loss. Elevated CRP, as a marker of systemic inflammation, is associated with reduced appetite, inadequate nutritional intake, and hypermetabolism; in parallel, decreased albumin levels reflect impaired nutritional status [32]. Indeed, the GLIM criteria emphasize the role of inflammation in the diagnosis of malnutrition and highlight its association with low albumin levels. The vicious cycle driven by inflammation not only contributes to malnutrition but also promotes muscle proteolysis, thereby accelerating the development of sarcopenia [33]. Therefore, the CRP/albumin ratio (CAR) emerges as a combined biomarker that simultaneously reflects both the inflammatory response and nutritional status.

CAR has been evaluated as a prognostic marker in various patient populations. In critically ill patients, elevated CAR levels have been associated with 28-day mortality [16]. In patients with heart failure, a higher CAR has been linked to increased mortality, more frequent hospitalizations, and a higher risk of advanced heart failure [15]. A recent meta-analysis including data from 2954 stroke patients demonstrated that elevated CAR values are a prognostic factor for poor functional outcomes and mortality [31]. Furthermore, in stroke patients who underwent recanalization therapy, high CAR levels were reported to be associated with intracranial hemorrhage and unfavorable functional outcomes [34,35]. However, no study has directly examined the relationship between CAR and malnutrition and sarcopenia in stroke patients. Our study is the first to present in the literature and offers a unique contribution in this respect.

In our study, the SARC-F questionnaire was used for sarcopenia screening, as recommended in the literature. Handgrip strength measurement was subsequently performed in patients identified as being at risk for sarcopenia to evaluate muscle strength [22]. Probable sarcopenia was detected in 19.3% of the patients. A meta-analysis reported the prevalence of sarcopenia as 15.8% prior to stroke and 29.5% within the first 10 days after stroke [36]. Our findings appear to be consistent with these reported rates. It is well established that inflammatory conditions accelerate muscle catabolism and contribute to muscle loss. Elevated levels of C-reactive protein (CRP), a marker of inflammation, have been reported to be associated with sarcopenia [37]. Furthermore, individuals with sarcopenia have been shown to exhibit lower serum albumin levels compared to those without sarcopenia. Therefore, the CRP-to-albumin ratio (CAR), which reflects both inflammatory and nutritional status simultaneously, may serve as a stronger biomarker for predicting sarcopenia. Indeed, Wong et al. reported that high CAR values were an independent predictor of low muscle mass in 91 hemodialysis patients [38].

In our study, the performance of CAR in predicting sarcopenia was evaluated using ROC analysis, and the cut-off value was determined as 0.320 (sensitivity 81.6%; specificity 71.7%). Additionally, in our study, the CAR cut-off value for predicting malnutrition risk was determined as 0.306 (sensitivity 68.8%; specificity 75.2%). Kaya et al. reported a CAR threshold of ≥0.86 in a study involving 154 geriatric patients, with a sensitivity of 48.4% and specificity of 71.7% for predicting nutritional status. However, sarcopenia was not assessed in that study [39]. In another study conducted in patients with chronic obstructive pulmonary disease (COPD), the CAR cut-off value for predicting malnutrition risk was calculated as 3.26. This high value may be attributed to the presence of pneumonia in approximately one-fifth of the patients, as well as the inclusion of individuals with renal failure [40]. Our study provides specific data on acute stroke patients, supported by well-defined inclusion criteria designed to minimize potential confounding conditions that could affect CRP and albumin levels.

CAR has been utilized in various patient populations both as a prognostic factor and as a marker for predicting malnutrition. In a study by Mocellin et al., conducted on patients with colorectal cancer, supplementation with polyunsaturated fatty acids (PUFAs), known for their immunomodulatory effects, was found to reduce CAR levels. In the group with decreased CAR levels, weight gain was observed after 9 weeks, whereas weight loss occurred in the group with increased CAR levels [41]. These findings suggest that elevated CAR is associated with poor nutritional status and that CAR may be a modifiable parameter through nutritional interventions. Similarly, in our study, elevated CAR was identified as an independent predictor of both malnutrition risk and sarcopenia.

Our study has several limitations. First, objective methods such as CT, DXA, or BIA were not used for the diagnosis of sarcopenia; therefore, the evaluation was limited to patients with probable sarcopenia. Second, although the study was conducted in two centers, larger, multicenter, and long-term prospective studies are needed to improve the generalizability of the findings. Nevertheless, the consistent predictive role of CAR in both malnutrition risk and probable sarcopenia observed in this study suggests a potential shared pathophysiological mechanism linking systemic inflammation, nutritional depletion, and muscle weakness in post-stroke patients. We hypothesize that CAR may serve as a practical biomarker integrating these processes, and future prospective trials are warranted to test this hypothesis [38,42].

## 5. Conclusions

In this study, CAR was identified as an independent predictor of both malnutrition risk and probable sarcopenia in patients with ischemic stroke. These findings suggest that CAR may reflect the combined impact of systemic inflammation, nutritional status, and muscle health. Although our results highlight the potential clinical relevance of CAR, further large-scale, multicenter, and prospective studies are needed before its routine use in early nutritional assessment can be recommended. These findings suggest that CAR is not only a marker of prognosis but also a practical and cost-effective biomarker that may reflect nutritional status and muscle health. Our results provide promising evidence for the potential routine use of CAR in the early nutritional assessment of stroke patients.

## Figures and Tables

**Table 1:** Baseline Demographic and Clinical Features According to Malnutrition Status.

Variable	Not at Risk of Malnutrition (n = 133)	At Risk of Malnutrition (n = 64)	*p*-Value
Age (years, mean ± SD)	66.63 ± 12.67	74.33 ± 11.04	**<0.001**
Sex (M/F)	74/59	37/27	0.893
NIHSS (mean ± SD)	3.97 ± 3.35	6.00 ± 4.27	**0.001**
BMI (kg/m^2^, mean ± SD)	29.21 ± 5.09	26.33 ± 3.99	**<0.001**
DM, n (%)	80 (60.2%)	33 (51.6%)	0.323
HT, n (%)	108 (81.2%)	49 (76.6%)	0.569
CAD, n (%)	60 (45.1%)	33 (51.6%)	0.485
HL, n (%)	105 (78.9%)	47 (73.4%)	0.495
AF, n (%)	32 (24.1%)	30 (46.9%)	**0.002**
Smoking, n (%)	33 (24.8%)	10 (15.6%)	0.201
CAR (mean ± SD)	0.364 ± 0.552	1.486 ± 1.935	**<0.001**

Patients were classified as “not at risk of malnutrition” (NRS-2002 < 3, n = 133) and “at risk of malnutrition” (NRS-2002 ≥ 3, n = 64). Abbreviations: M: Male; F: Female; NIHSS: National Institutes of Health Stroke Scale; BMI: Body Mass Index; DM: Diabetes Mellitus; HT: Hypertension; CAD: Coronary Artery Disease; HL: Hyperlipidemia; AF: Atrial Fibrillation; CAR: C-Reactive protein/Albumin Ratio.

**Table 2:** Univariate and multivariate logistic regression analysis of risk factors associated with malnutrition risk.

Variable	Univariate			Multivariate		
	OR	95% CI	*p*-Value	OR	95% CI	*p*-Value
Age (years)	1.06	1.03–1.09	**<0.001**	1.05	1.02–1.09	**0.003**
Sex (Male)	0.91	0.50–1.67	0.773	1.55	0.57–4.25	0.390
NIHSS	1.15	1.06–1.25	**<0.001**	1.11	1.01–1.23	**0.026**
Diabetes Mellitus	0.70	0.38–1.28	0.254	–	–	–
Hypertension	0.75	0.37–1.56	0.449	–	–	–
Coronary Artery Disease	1.29	0.71–2.35	0.396	–	–	–
Hyperlipidemia	0.73	0.36–1.48	0.389	–	–	–
Atrial Fibrillation	2.78	1.48–5.24	**0.001**	1.49	0.68–3.24	0.314
Smoking	0.56	0.25–1.22	0.147	–	–	–
BMI (kg/m^2^)	0.86	0.80–0.94	**<0.001**	0.89	0.82–0.97	**0.010**
CAR (CRP/Albumin ratio)	2.47	1.64–3.70	**<0.001**	2.13	1.39–3.26	**<0.001**

NIHSS = National Institutes of Health Stroke Scale; BMI = Body Mass Index; CAR = C-Reactive protein/Albumin Ratio.

**Table 3:** Baseline demographic and clinical variables in patients according to probable sarcopenia status.

Variable	No Probable Sarcopenia (n = 159)	Probable Sarcopenia (n = 38)	*p*-Value
Age (years, mean ± SD)	66.90 ± 12.35	78.47 ± 9.33	**<0.001**
Sex (Male/Female), n	91/68	20/18	0.607
NIHSS at admission (mean ± SD)	4.28 ± 3.63	6.08 ± 4.10	**0.007**
Diabetes Mellitus, n/total	94/159	19/38	0.362
Hypertension, n/total	124/159	33/38	0.268
Coronary Artery Disease, n/total	72/159	21/38	0.284
Hyperlipidemia, n/total	125/159	27/38	0.389
Atrial Fibrillation, n/total	45/159	17/38	0.055
Smoking, n/total	40/159	3/38	**0.027**
BMI (kg/m^2^, mean ± SD)	28.64 ± 4.93	26.72 ± 4.75	**0.004**
CAR (CRP/Albumin ratio, mean ± SD)	0.47 ± 0.89	1.81 ± 2.01	**<0.001**

NIHSS = National Institutes of Health Stroke Scale; BMI = Body Mass Index; CAR = C-Reactive protein/Albumin Ratio. Probable sarcopenia was defined as SARC-F ≥ 4 and low handgrip strength (<27 kg for men, <16 kg for women).

**Table 4:** Univariate and multivariate analysis of variables associated with probable sarcopenia.

Variable	Univariate			Multivariate		
	OR	95% CI	*p*-Value	OR	95% CI	*p*-Value
Age (years)	1.13	1.07–1.18	**<0.001**	1.12	1.05–1.18	**<0.001**
Sex (Male)	0.83	0.41–1.69	0.608	1.68	0.62–4.53	0.308
NIHSS	1.12	1.03–1.23	**0.010**	1.16	1.04–1.31	**0.011**
Diabetes Mellitus	0.69	0.34–1.41	0.309	-	-	-
Hypertension	1.86	0.68–5.13	0.229	-	-	-
Coronary Artery Disease	1.49	0.73–3.04	0.270	-	-	-
Hyperlipidemia	0.67	0.30–1.48	0.320	-	-	-
Atrial Fibrillation	2.05	0.99–4.24	0.053	-	-	-
Smoking	0.25	0.07–0.87	**0.030**	0.60	0.11–3.33	0.563
BMI (kg/m^2^)	0.92	0.85–0.99	**0.035**	0.98	0.89–1.08	0.676
CAR (CRP/Albumin ratio)	1.96	1.45–2.64	**<0.001**	1.76	1.22–2.53	**0.003**

NIHSS = National Institutes of Health Stroke Scale; BMI = Body Mass Index; CAR = C-Reactive protein/Albumin Ratio.

## Data Availability

The data presented in this study are available on request from the corresponding author, the data are not publicly available due to privacy or ethical restrictions.

## Supplementary Materials

The following supporting information can be downloaded at: https://www.mdpi.com/article/10.3390/jcm14196804/s1, Table S1: Collinearity Diagnostics for Malnutrition Model; Table S2: Collinearity Diagnostics for Probable Sarcopenia Model.

## References

[1] Cheng Y., Lin Y., Shi H., Cheng M., Zhang B., Liu X., Shi C., Wang Y., Xia C., Xie W. (2024). Projections of the Stroke Burden at the Global, Regional, and National Levels up to 2050 Based on the Global Burden of Disease Study 2021. J. Am. Heart Assoc..

[2] Feigin V.L., Brainin M., Norrving B., Martins S.O., Pandian J., Lindsay P., Grupper M.F., Rautalin I. (2025). World Stroke Organization: Global Stroke Fact Sheet 2025. Int. J. Stroke.

[3] Mosselman M.J., Kruitwagen C.L.J.J., Schuurmans M.J., Hafsteinsdóttir T.B. (2013). Malnutrition and risk of malnutrition in patients with stroke: Prevalence during hospital stay. J. Neurosci. Nurs..

[4] Crary M.A., Carnaby-Mann G.D., Miller L., Antonios N., Silliman S. (2006). Dysphagia and Nutritional Status at the Time of Hospital Admission for Ischemic Stroke. J. Stroke Cerebrovasc. Dis..

[5] Crary M.A., Humphrey J.L., Carnaby-Mann G., Sambandam R., Miller L., Silliman S. (2013). Dysphagia, nutrition, and hydration in ischemic stroke patients at admission and discharge from acute care. Dysphagia.

[6] Davis J.P., Wong A.A., Schluter P.J., Henderson R.D., O’Sullivan J.D., Read S.J. (2004). Impact of premorbid undernutrition on outcome in stroke patients. Stroke.

[7] Mas M.F., González J., Frontera W.R. (2020). Stroke and sarcopenia. Curr. Phys. Med. Rehabil. Rep..

[8] Sabbouh T., Torbey M.T. (2018). Malnutrition in Stroke Patients: Risk Factors, Assessment, and Management. Neurocrit. Care.

[9] Kim S.Y., Cho W.S., Park C.B., Kim B.G. (2024). Effect of Sarcopenia on Functional Recovery in Acute Stroke Patients Admitted for Standard Rehabilitation Program. Medicina.

[10] Lambertsen K.L., Finsen B., Clausen B.H. (2019). Post-stroke inflammation—Target or tool for therapy?. Acta Neuropathol..

[11] Soeters P.B., Wolfe R.R., Shenkin A. (2019). Hypoalbuminemia: Pathogenesis and Clinical Significance. JPEN J. Parenter. Enter. Nutr..

[12] Thuemmler R.J., Pana T.A., Carter B., Mahmood R., Bettencourt-Silva J.H., Metcalf A.K., Mamas M.A., Potter J.F., Myint P.K. (2024). Serum Albumin and Post-Stroke Outcomes: Analysis of UK Regional Registry Data, Systematic Review, and Meta-Analysis. Nutrients.

[13] Den Hertog H.M., Van Rossum J.A., Van Der Worp H.B., Van Gemert H.M.A., De Jonge R., Koudstaal P.J., Dippel D.W., PAIS investigators (2009). C-reactive protein in the very early phase of acute ischemic stroke: Association with poor outcome and death. J. Neurol..

[14] Bian J., Guo S., Huang T., Li X., Zhao S., Chu Z., Li Z. (2023). CRP as a potential predictor of outcome in acute ischemic stroke. Biomed. Rep..

[15] Kurniawan R.B., Oktafia P., Saputra P.B.T., Purwati D.D., Saputra M.E., Maghfirah I., Faizah N.N., Oktaviono Y.H., Alkaff F.F. (2024). The roles of C-reactive protein-albumin ratio as a novel prognostic biomarker in heart failure patients: A systematic review. Curr. Probl. Cardiol..

[16] Park J.E., Chung K.S., Song J.H., Kim S.Y., Kim E.Y., Jung J.Y., Kang Y.A., Park M.S., Kim Y.S., Chang J. (2018). The C-Reactive Protein/Albumin Ratio as a Predictor of Mortality in Critically Ill Patients. J. Clin. Med..

[17] Liao C.-K., Yu Y.-L., Lin Y.-C., Hsu Y.-J., Chern Y.-J., Chiang J.-M., You J.-F. (2021). Prognostic value of the C-reactive protein to albumin ratio in colorectal cancer: An updated systematic review and meta-analysis. World J. Surg. Oncol..

[18] Liu Z.Y., Tang J.N., Cheng M.D., Jiang L.Z., Guo Q.Q., Zhang J.C., Zhang Z.L., Song F.H., Wang K., Fan L. (2021). C-reactive protein-To-serum albumin ratio as a novel predictor of long-Term outcomes in coronary artery disease patients who have undergone percutaneous coronary intervention: Analysis of a real-world retrospective cohort study. Coron. Artery Dis..

[19] Tonet E., Campana R., Caglioni S., Gibiino F., Fiorio A., Chiaranda G., Zagnoni S., Casella G., Campo G. (2021). Tools for the Assessment of the Malnutrition Status and Possible Interventions in Elderly with Cardiovascular Diseases. J. Clin. Med..

[20] Han P., Yu H., Ma Y., Kang L., Fu L., Jia L., Chen X., Yu X., Hou L., Wang L. (2017). The increased risk of sarcopenia in patients with cardiovascular risk factors in Suburb-Dwelling older Chinese using the AWGS definition. Sci. Rep..

[21] Kondrup J., Allison S.P., Elia M., Vellas B., Plauth M., Educational and Clinical Practice Committee, European Society of Parenteral and Enteral Nutrition (ESPEN) (2003). ESPEN guidelines for nutrition screening 2002. Clin. Nutr..

[22] Cruz-Jentoft A.J., Bahat G., Bauer J., Boirie Y., Bruyère O., Cederholm T., Cooper C., Landi F., Rolland Y., Sayer A.A. (2019). Sarcopenia: Revised European consensus on definition and diagnosis. Age Ageing.

[23] Malmstrom T.K., Miller D.K., Simonsick E.M., Ferrucci L., Morley J.E. (2016). SARC-F: A symptom score to predict persons with sarcopenia at risk for poor functional outcomes. J. Cachexia Sarcopenia Muscle.

[24] Burgos R., Bretón I., Cereda E., Desport J.C., Dziewas R., Genton L., Gomes F., Jésus P., Leischker A., Muscaritoli M. (2018). ESPEN guideline clinical nutrition in neurology. Clin. Nutr..

[25] Gomes F., Emery P.W., Weekes C.E. (2016). Risk of Malnutrition Is an Independent Predictor of Mortality, Length of Hospital Stay, and Hospitalization Costs in Stroke Patients. J. Stroke Cerebrovasc. Dis..

[26] Foley N.C., Salter K.L., Robertson J., Teasell R.W., Woodbury M.G. (2009). Progress Review Which Reported Estimate of the Prevalence of Malnutrition After Stroke Is Valid?. Stroke.

[27] Çoban E., Soysal A. (2021). The Profile of a Neurology Clinic and Malnutrition Awareness. Turk. J. Neurol..

[28] Liu Q., Shi K., Wang Y., Shi F.D. (2023). Neurovascular Inflammation and Complications of Thrombolysis Therapy in Stroke. Stroke.

[29] Pawluk H., Woźniak A., Tafelska-Kaczmarek A., Kosinska A., Pawluk M., Sergot K., Grochowalska R., Kołodziejska R. (2025). The Role of IL-6 in Ischemic Stroke. Biomolecules.

[30] Chen L., Wang M., Yang C., Wang Y., Hou B. (2023). The role of high-sensitivity C-reactive protein serum levels in the prognosis for patients with stroke: A meta-analysis. Front. Neurol..

[31] Yang J., Chen Y., Wan J., Li F., Yang X., Shen B., Li N., Didi J., Zhou L., Zhang Y. (2025). Prognostic value of the C-reactive protein to albumin ratio in patients with stroke: A meta-analysis. Sci. Rep..

[32] Pourhassan M., Cederholm T., Donini L.M., Poggiogalle E., Schwab U., Nielsen R.L., Andersen A.L., Małgorzewicz S., Volkert D., Wirth R. (2023). Severity of Inflammation Is Associated with Food Intake in Hospitalized Geriatric Patients-A Merged Data Analysis. Nutrients.

[33] Chon J., Soh Y., Shim G.Y. (2024). Stroke-Related Sarcopenia: Pathophysiology and Diagnostic Tools. Brain Neurorehabil..

[34] de Liyis B.G., Ardhaputra G.Y.B., Liyis S., Wihandani D.M., Siahaan Y.M.T., Pinatih K.J.P. (2024). High C-Reactive Protein/Albumin Ratio Predicts Mortality and Hemorrhage in Stroke Patients Undergoing Mechanical Thrombectomy: A Systematic Review and Meta-Analysis. World Neurosurg..

[35] Xu T., Xia L., Wu Y., Xu Y., Xu X., Zhang W., Zhou C., Fu F., Cao Y., Han Z. (2023). High ratio of C-reactive protein to albumin is associated with hemorrhagic transformation and poor functional outcomes in acute ischemic stroke patients after thrombolysis. Front. Aging Neurosci..

[36] Inoue T., Ueshima J., Kawase F., Kobayashi H., Nagano A., Murotani K., Saino Y., Maeda K. (2023). Trajectories of the Prevalence of Sarcopenia in the Pre- and Post-Stroke Periods: A Systematic Review. Nutrients.

[37] Bano G., Trevisan C., Carraro S., Solmi M., Luchini C., Stubbs B., Manzato E., Sergi G., Veronese N. (2017). Inflammation and sarcopenia: A systematic review and meta-analysis. Maturitas.

[38] Wong T.-C., Su H.-Y., Chen Y.-T., Wu P.-Y., Chen H.-H., Chen T.-H., Hsu Y.-H., Yang S.-H. (2016). Ratio of C-Reactive Protein to Albumin Predicts Muscle Mass in Adult Patients Undergoing Hemodialysis. PLoS ONE.

[39] Kaya T., Ulaş S.B., Nalbant A., Yıldırım I., İşSever K., Karacaer C., Bilgin C., Vatan A., Acar T., Acar B.A. (2024). C-reactive protein/albumin ratio as a novel predictor for nutritional status of geriatric patients. Brain Behav..

[40] Baldemir R., Öztürk A., Eraslan Doganay G., Cirik M.O., Alagoz A. (2022). Evaluation of Nutritional Status in Hospitalized Chronic Obstructive Pulmonary Disease Patients and Can C-reactive Protein-to-Albumin Ratio Be Used in the Nutritional Risk Assessment in These Patients. Cureus.

[41] Mocellin M.C., Pastore E Silva J.D.A., Camargo C.D.Q., Fabre M.E.D.S., Gevaerd S., Naliwaiko K., Moreno Y.M., Nunes E.A., Trindade E.B. (2013). Fish oil decreases C-reactive protein/albumin ratio improving nutritional prognosis and plasma fatty acid profile in colorectal cancer patients. Lipids.

[42] Tur K., Güçlü A. (2024). Independent Association Between Malnutrition Inflammation Score and C Reactive Protein/Albumin Ratio in Hemodialysis Patients. J. Inflamm. Res..

