# Supplementary material for: Predictive Value of C-Reactive Protein/Albumin Ratio (CAR) for Malnutrition and Sarcopenia in Acute Ischemic Stroke Patients
Source: J Clin Med. 2025 Sep 26;14(19):6804. doi: 10.3390/jcm14196804 (PMC12524821; doi:10.3390/jcm14196804)
Supplement: Supplementary file 1 [file jcm-14-06804-s001.zip › jcm-3878411-supplementary.pdf]

Supplementary Table S1: Collinearity Diagnostics for Malnutrition Model

Variance inflation factor (VIF) and tolerance values were calculated for all independent variables included in the logistic regression model for malnutrition. All variables had VIF values < 2 and tolerance values > 0.1, indicating no evidence of multicollinearity.

Variable	VIF	Tolerance
Age	1.7	0.59
Sex	1.3	0.77
NIHSS	1.13	0.89
Diabetes Mellitus	1.18	0.85
Hypertension	1.28	0.78
Coronary Artery Disease	1.19	0.84
Hyperlipidemia	1.13	0.89
Atrial Fibrillation	1.22	0.82
Smoking	1.64	0.61
BMI (kg/m^2)	1.26	0.79
CAR (CRP/Albumin ratio)	1.25	0.8

NIHSS = National Institutes of Health Stroke Scale; BMI = Body Mass Index; CAR = C-Reactive protein/Albumin Ratio.

Supplementary Table S2: Collinearity Diagnostics for Probable Sarcopenia Model

Variance inflation factor (VIF) and tolerance values were calculated for all independent variables included in the logistic regression model for probable sarcopenia. All variables had VIF values < 2 and tolerance values > 0.1, indicating no evidence of multicollinearity.

Variable	VIF	Tolerance
Age	1.7	0.59
Sex	1.3	0.77
NIHSS	1.13	0.89
Diabetes Mellitus	1.18	0.85
Hypertension	1.28	0.78
Coronary Artery Disease	1.19	0.84
Hyperlipidemia	1.13	0.89
Atrial Fibrillation	1.22	0.82
Smoking	1.64	0.61
BMI (kg/m^2)	1.26	0.79
CAR (CRP/Albumin ratio)	1.25	0.8

NIHSS = National Institutes of Health Stroke Scale; BMI = Body Mass Index; CAR = C-Reactive protein/Albumin Ratio.
